# The Determinants of Mental Health Literacy among Young Adolescents in Malaysia

**DOI:** 10.3390/ijerph19063242

**Published:** 2022-03-09

**Authors:** Sarbhan Singh, Rafdzah Ahmad Zaki, Nik Daliana Nik Farid, Kushilpal Kaur

**Affiliations:** 1 Institute for Medical Research (IMR), National Institutes of Health (NIH), Ministry of Health Malaysia, Shah Alam 40170, Malaysia; 2 Department of Social and Preventive Medicine, Faculty of Medicine, University of Malaya, Kuala Lumpur 50603, Malaysia; drrafdzah@um.edu.my (R.A.Z.); daliana@um.edu.my (N.D.N.F.); 3 Department of Psychiatry, Hospital Selayang, Ministry of Health Malaysia, Batu Caves 68100, Malaysia; kushil67@hotmail.com

**Keywords:** mental health literacy, adolescents, mental health disorders

## Abstract

Mental health literacy (MHL) is an established multifaceted concept that comprises mental health knowledge, help-seeking, and stigma. Adequate MHL (i.e., the ability to correctly recognize mental health disorders alongside having the intention to seek help) is able improve mental health outcomes among individuals. This study aims to examine the determinants of MHL among young Malaysian adolescents. A cross-sectional study was conducted among 1400 adolescents between 13 and 14 years old from nine national secondary schools in Selangor state, Malaysia. Sociodemographic determinants assessed included gender, age, ethnicity, smoking status, alcohol consumption, history of being bullied, feeling lonely, parental marital status, and parental income which were assessed using the Global School Based Student Health Survey. MHL was assessed using the Mental Health Literacy and Stigma questionnaire. Several factors were significantly associated with adequate levels of MHL following multivariate analysis, such as being female (AOR = 1.68; 95% CI 1.12, 2.52), older adolescents (AOR = 1.56; 95% CI 1.07, 2.30), not smoking (AOR = 1.99; 95% CI 1.20, 4.26), not consuming alcohol (AOR = 1.23; 95% CI 1.18, 2.41), and not feeling lonely (AOR = 1.25; 95% CI 1.06, 1.85). Addressing these determinants could be key in assisting the development of policies and programs to prevent mental health disorders among adolescents, which are currently on the rise.

## 1. Introduction

Mental health literacy (MHL) is a multifaceted concept first introduced by Jorm et al. in 1996, which emphasizes improving mental health knowledge, help-seeking, and addressing mental health stigma, with the aim of improving mental health [1]. The mental health knowledge component of MHL focuses on improving the ability of individuals to correctly recognize mental health disorders early on. In addition, this component also centers around the knowledge regarding mental health first aid, interventions, and appropriate preventive measures that could essentially improve mental health outcomes [2]. The help-seeking component of MHL promotes appropriate help-seeking intentions, selection of suitable sources of help, and identification and elimination of barriers to help-seeking [3,4]. The stigma component of MHL covers a wide spectrum of stigma related to mental health disorders (i.e., stigmatizing attitudes, perceived/personal stigma, and social distancing) with the aim of destigmatizing mental health disorders [3,4]. As the components of MHL are often interrelated, they need to be assessed in a holistic manner to be able to subsequently improve the overall levels of MHL [4,5,6].

Due to the extensive and comprehensive nature of the MHL concept, it has gained the attention of many researchers globally, and as a result, many studies have examined this concept in several ways, including assessing the levels of MHL and determining the association/relationships between MHL and mental health disorders [4,5,6,7]. Levels of MHL tend to vary by region; for example, higher levels of MHL are frequently observed within the western population and in more developed nations [4,5,6,7]. In addition, evidence in the literature also reports that individuals with low or inadequate levels of MHL tend to have poorer mental health outcomes and an increased risk of developing mental health disorders [7]. These findings are attributed to the fact that individuals with low MHL would not be able to recognize mental health disorders in a timely fashion, which would subsequently delay the process of help-seeking [8,9]. Furthermore, low MHL also results in higher mental health stigma, which could ultimately be a barrier to help-seeking [10]. From the existing evidence in the literature, it is clear that increasing MHL is key to improve mental health outcomes [8,9,10]. One approach to improve MHL is to identify the determinants of MHL, as this would then enable decision makers to subsequently address these determinants to ensure better MHL, which would result in more positive mental health outcomes. Several studies have identified determinants of MHL; the majority of these studies have reported higher levels of MHL among females, older people, higher educational levels, and higher family income [11,12,13,14,15,16]. However, most of these studies have been conducted among the adult population in developed nations. Therefore, it would be inappropriate to generalize the existing findings to younger populations residing in developing nations due to underlying variations in sociodemographic characteristics.

In Malaysia, low levels of MHL have been reported among young adolescents, wherein only a minority (3%) of adolescents were classified as having adequate MHL (i.e., the ability to correctly recognize mental health disorders alongside having the intention to seek help) [17]. More concerning is that adolescents with low levels of MHL have an increased risk of developing mental health disorders [7]. A report released by the National Health Morbidity Survey (NHMS) in 2017 revealed that the prevalence of mental health disorders among Malaysian children and young adolescents aged 5 to 15 showed an increasing trend, escalating from 19.4% in 1996 to 22.6% in 2017 [18]. With the rising prevalence of mental health disorders and more severe forms of mental health diseases affecting younger adolescents between 13 and 14 years old, it is important to improve MHL early on by identifying the determinants of MHL unique to this population group. However, to date in Malaysia, there are no published studies on the determinants of MHL among young adolescents. With low MHL being viewed as a global public health concern, many developed countries (i.e., Australia and the USA) have already extensively studied the determinants of MHL and subsequently instituted nationwide MHL programs; however, developing countries are currently still far behind in this context [14,19]. Due to this, the aim of this study is to examine the determinants of the various components of MHL (i.e., ability to recognize mental health disorders, intention to seek help, and overall adequacy of MHL) among young adolescents in Malaysia. We believe the findings from this study will provide valuable data on the determinants of MHL, which could assist decision makers in making evidence informed decisions relating to the development of mental health policies and programs to improve mental health among young adolescents.

## 2. Materials and Methods

### 2.1. Study Design and Sampling Strategy

A cross-sectional study was conducted involving students from National Secondary Schools in Selangor State, which is located in Peninsular Malaysia, from August to November 2019. Using a two-stage sampling method, nine schools were randomly selected from the list of national secondary schools in Selangor. At the school level, lower secondary students (Form 1 and 2) were sample randomly to participate in this study. Random selection was performed using an Excel RAND function. A list of 278 randomly generated numbers was obtained from the Excel RAND formula, then the random values generated were ordered and the first 9 schools in the sorted list were selected. The similar process was repeated at the school level wherein around 155 students were selected from each school. The justification to study adolescents aged between 13 and 14 years is due to the rising prevalence of mental health disorder among young adolescent whose severity tends to increase with advancing ages [18]. In addition, these students were not scheduled for major examinations; therefore, changes in classroom schedule would not negatively affect their academic work.

### 2.2. Sample Size Calculation

G* power software version 3 was used for sample size calculation, wherein the following parameters in G* power software were used [20]: (a) two tail, (b) adjusted odds ratio (AOR) for gender and adequate MHL was set at 2.18 (95% confidence interval: 1.82–2.61) based on a study by Kaneko and Motohashi (2007) [21], (c) Pr (Y = 1|X = 1)H0, the probability of an outcome (adequate MHL) among participants without exposure (males) 0.25 based on a study by Kaneko and Motohashi (2007) [21], (d) alpha set at 0.05, (e) power set at 80%, (f) R2 other exposure variable set as zero, as AOR was used to indicate that effects from all other covariates were adjusted and accounted for, (g) binomial distribution was selected as the exposure variable, which was gender, as a binomial variable and (f) X parm, the proportion of those with the outcome (adequate MHL) who had exposure (females) = 0.92 [21]. The sample size required was 1396 (Appendix A).

### 2.3. Data Collection

Data collection was conducted at each respective school at a date chosen by the school. Before data collection, students were assembled and were briefed on the purpose of this study and their rights as respondents by the researcher. Following this, students were given a research information sheet and consent form, which the students had to read and bring home to give their guardians or parents to read and sign if they agreed to participate. Students were advised to return the consent forms to the respective school counselors within 1 to 2 days. After 3 days, the respective school counselor informed the researcher of the number of consent forms returned. A subsequent date was set, at which time, the researcher distributed the study questionnaire to the participants in the respective schools. All questionnaires were coded and had no personal identifiers to ensure the confidentiality of participants was maintained. The average time taken to complete the questionnaire was 15 min. After the session, the researcher collected all the questionnaires from the participants. To minimize missing data, firstly, the researcher advised all participants to check their questionnaire at the end to ensure all questions were answered; secondly, the researcher checked all the returned questionnaires immediately upon collection to detect any missing data. The same procedures as above were repeated for the distribution and collection of questionnaires in each school. Parental consent forms were distributed to 1400 students who met the inclusion criteria (i.e., lower secondary school students ages between 13 and 14 years). In total, 1400 participants consented to the study and were included in the analysis. The data set is available at the Supplementary Materials Section.

### 2.4. Measures

#### 2.4.1. Global School Based Student Health Survey

Participant sociodemographic characteristics were assessed using the Global School Based Student Health Survey [22]. These variables were assessed using a binary level response option for gender (male/female), age (13/14 years), ethnicity (Malay/Non-Malay), smoking status (smoker/nonsmoker), alcohol consumptions (yes/no), history of being bullied (yes/no), feeling lonely (yes/no), parental marital status (married and living together/divorced or separated) and parental income (low income of less than MYR 3000 per month/ moderate to high income of equal to or more than MYR 3000 per month)

#### 2.4.2. Mental Health Literacy and Stigma Questionnaire

MHL was assessed using the Mental Health Literacy (MHL) and Stigma questionnaire which has been adopted, translated, and widely used in many studies across various countries to assess MHL among adolescents [1,2]. Depression was selected as the mental health disorder in this study, because depression is highly prevalent and is the most common mental health disorder affecting 1 in 5 adolescents in Malaysia [18]. The MHL and stigma questionnaire presented a scenario of a depressed person based on the Diagnostic and Statistical Manual of Mental Disorders (DSM) criteria as follows:

“*Ali is a 15-year-old who has been feeling unusually sad and miserable for the last few weeks. He is tired all the time and has trouble sleeping at night. Ali doesn’t feel like eating and has lost weight. He can’t keep his mind on his studies and his marks have dropped. He puts off making any decisions and even day-to-day tasks seem too much for him. His parents and friends are very concerned about him*.”

Then, participants were presented with several questions in each section relating to each component of MHL, which were based on the person described in the vignette. There were a total of 90 items on the self-administered questionnaire, with responses based on a 3-point Likert scale, of which 55 items related to the knowledge component of MHLS (i.e., knowledge of recognition of the disorder, first aid, interventions, and preventions for mental disorders), while the help-seeking and stigma (i.e., personal/perceived stigma and social distancing) components comprised 16 and 19 statements, respectively. In this study the knowledge and help-seeking components of MHL were determined by assessing the participant’s ability to correctly recognize depression and their intention to seek help. To assess participant’s ability to correctly recognize depression, respondents were asked the following question. “What, if anything, do you think is wrong with Ali?” The responses were composed of several terms or expressions, including “depression”, “schizophrenia”, “psychosis”, “cancer”, “mental illness”, “psychological, mental, emotional problem”, “bulimia”, “stress”, “substance abuse”, “age crisis”, “has a problem”, and “nothing”. To assess the help-seeking intentions of participant’s respondents were asked the following question “If you had a problem right now like Ali’s would you go for help?” For this questions, participants were supposed to answer in the following response format: “agree”, “disagree”, or “neutral”. A correct recognition of depression was considered if participants label the vignette as having depression, having a mental illness, and/or psychological, mental, emotional problem. Intention to seek help was considered positive if the participants agreed they would seek help if they had a similar problem to that described in the vignette. Subsequently, the variables relating to the ability to correctly recognize depression and the intention to seek help were combined to generate a composite variable that represented the adequacy of MHL, wherein participants who were able to correctly recognize depression alongside an intention to seek help were classified to have adequate levels of MHL [7]. The Mental Health Literacy (MHL) and Stigma questionnaire is a valid and reliable instrument for use among young Malaysia adolescents to examine MHL [23]. The Cronbach’s alpha for the knowledge and help-seeking components of the MHL and Stigma questionnaire was reported to be 0.71 and 0.76, respectively, in this study.

### 2.5. Analytic Approach

Data were analyzed using Statistical Program for the Social Sciences (SPSS) version 24.0. Before performing any statistical analysis, double data entry was performed to ensure the accuracy of the data and the minimization of error, and data were checked for missing data and abnormal values. No missing values were present. The descriptive analysis included frequency and percentages. To examine the determinants of MHL, a multivariate binary logistic regression analysis was performed. Variables with *p* < 0.25 of univariate analysis, with no evidence of multicollinearity and interaction, were considered significant and included in the multivariable model [24]. Multicollinearity was examined using the Cramer’s V, wherein any two variables with a Cramer’s V value of >0.3 were considered to have evidence of multicollinearity [24]. Evidence of interaction was examined using the test of the interaction function in SPSS, whereby a significant value (*p* < 0.05) indicates that interaction was present. There was no evidence of multicollinearity or interaction among the independent variables (Appendix B).

### 2.6. Ethics

The study was registered with the National Medical Research Register (NMRR-18-719-40569). Ethics approval was obtained from the University of Malaya Research Ethical Committee (UM.TNC 2/UMREC). Permission to use the school was approved by the Malaysian Ministry of Education, Selangor State Education Department, and the respective school principals. Written consent was obtained from the participant’s guardians. Permission to use the study instruments was obtained from the original authors. The data from this study are available in the supplementary file.

## 3. Results

A total of 1400 adolescents participated in this study, of which 676 (48.3%) and 724 (51.7%) were male and female, respectively. The age distribution was almost similar with 687 (49.1%) and 713 (50.9%) being 13 and 14 years old, respectively. The majority of the participants were Malay (*n* = 928, 66.3%). In total, there were 79 (5.6%) and 116 (8.3%) participants who smoked and consumed alcohol, respectively. A history of being bullied or feeling lonely was reported by 165 (11.8%) and 566 (4.0%) participants, respectively. The majority of the participants came from intact families (*n* = 1242, 88.7%) with a low parental income level (*n* = 970, 69.3%). With regard to the level of MHL among the participants, despite the majority of participants having the intention to seek help (*n* = 1139, 81.4%), only a few were able to correctly recognize depression (*n* = 128, 9.1%). Therefore, this resulted in high levels of inadequate MHL among participants in this study (*n* = 1278, 91.3%) as shown in Table 1.

Following multivariate analysis, several factors were significantly associated with MHL (Table 2, Table 3 and Table 4). With regard to factors associated with correct recognition of depression, the multivariate analysis found that being female (AOR = 1.74; 95% CI 1.17, 2.59), older adolescents (AOR = 1.54; 95% CI 1.06, 2.24), not smoking (AOR = 1.61; 95% CI 1.10, 3.57), not consuming alcohol (AOR = 1.31; 95% CI 1.06, 2.53), and not feeling lonely (AOR = 1.22; 95% CI 1.13, 1.79) were significantly associated with correct recognition of depression as shown in Table 2. The multivariate analysis to determine the factors associated with intention to seek help found that being female (AOR = 1.42; 95% CI 1.06, 1.89), older adolescents (AOR = 1.28; 95% CI 1.05, 1.69), not smoking (AOR = 1.68; 95% CI 1.01, 2.84), not consuming alcohol (AOR = 1.65; 95% CI 1.03, 2.73), and not feeling lonely (AOR = 1.32; 95% CI 1.02, 1.75) were significantly associated with the intention to seek help as shown in Table 3. Similarly, factors such as being female (AOR = 1.68; 95% CI 1.12, 2.52), older adolescents (AOR = 1.56; 95% CI 1.07, 2.30), not smoking (AOR = 1.99; 95% CI 1.20, 4.26), not consuming alcohol (AOR = 1.23; 95% CI 1.18, 2.41), and not feeling lonely (AOR = 1.25; 95% CI 1.06, 1.85) were reported to be significantly associated with adequate MHL as shown in Table 4.

## 4. Discussion

This study examined the sociodemographic determinants of MHL among young Malaysian adolescents. More specifically this study provided evidence on factors that influenced the various components of MHL, which included the knowledge component (i.e., the ability to correctly recognize a mental health disorder), the help-seeking component (i.e., the intention to seek help for an underlying mental health disorder), and the overall adequacy of MHL (i.e., the ability to correctly recognize a mental health disorder alongside having the intention to seek help).

This study reports that gender was a significant determinant influencing MHL among adolescents. Females demonstrated a higher ability to correctly recognize mental health disorders alongside having higher intentions to seek help and overall adequate MHL levels compared to their male counterparts. Similar findings have been observed among adolescents in the USA, Europe, Australia, and Hong Kong [25,26,27,28,29,30]. The effect of gender on MHL can be attributed to the manner in which symptoms of mental health disorders are identified and perceived across both genders. For example, males tend to use more general terms such as mental illness rather than specific terminologies (i.e., depression) in the process of identification of mental disorders, which could result in inaccurate identification of specific mental health disorders. It has also been reported that younger males fail to recognize symptoms of mental health disorders, as they perceive these symptoms to be a reaction to an external cause such as family problems or peer pressure rather than acknowledging it as a mental health disorder [27,28,29]. The higher intention to seek help among females can be mediated by the increased ability to correctly recognize a mental health disorder, which would subsequently increase the intention to seek help. Furthermore, a higher degree of externalization of mental health symptoms and poor symptom identification among males would further result in lower help-seeking intentions.

This study also reported advancing age as a significant determinant that improved MHL among adolescents. In this study, adolescents aged 14 years demonstrated better ability to correctly recognize mental health disorders, had higher intention to seek help, and greater incidence of adequate MHL levels of compared to younger adolescents. Our findings are consistent with previous studies conducted in China, Australia and the USA, which reported higher MHL among older population groups [25,31,32]. The effect of age on MHL can be mediated in several ways. With the advancement in age, individuals tend to gain more knowledge and exposure through higher education, which would in turn increase their level of awareness regarding MHL [33]. It also could be possible that an older adolescent is able to acquire knowledge on MHL easily through several avenues such as school-based mental health programs or gaining information regarding MHL from social media [31,32]. These reasons could result in older adolescents being more well informed about MHL compared to their younger peers.

In addition, to gender and age being significant determinants of MHL, this study also reported a significant association between smoking and alcohol consumption with MHL. Interestingly, we found that adolescents who did not smoke or consume alcohol tended to have higher MHL levels. These findings do not come as a surprise, because individuals may resort to substance abuse as a form of coping mechanism to deal with mental health disorders [34,35]. These negative forms of coping mechanism are adopted as a result of failure to recognize or acknowledge mental health disorders, which would result in delayed help-seeking [34]. Although there are limited data to support the effect of smoking and alcohol consumption on MHL specifically, there are several published studies that have reported the negative effects of substance abuse (i.e., smoking/alcohol consumption) on health literacy [36,37,38,39].

In this study, MHL levels were also found to be higher among adolescents who were not lonely. Similar findings were reported among young adults in Thailand and China, wherein significant negative correlation was reported between MHL and loneliness [40,41]. Individuals who are lonely tend to have restricted social networks, engage in fewer social activities, have limited social contacts, and lower levels of social support [42]. All these factor combined would subsequently limit the possibility of these individuals of gaining awareness of, understanding, adopting, or practicing the concept of MHL in their lives [41].

Being the first study to examine the determinants of MHL among young Malaysia adolescents, there were several limitations. Among these, we include the inability to generalize the study findings to older adolescents in Malaysia, as this study focused on young adolescents. Nevertheless, the approach used in this study could be replicated among older adolescents in future studies. Our findings regarding age being a significant determinant of MHL must be interpreted with caution due to the small difference in the participants age groups (1-year difference); a wider age group range might provide more understanding on the effects of age on MHL, which could be a direction for future research.

## 5. Conclusions

This study showed that the various components of MHL and the overall adequacy of MHL levels were associated with gender, age, smoking, alcohol consumption, and loneliness. Therefore, in order to improve MHL among young adolescents, it is crucial that MHL policies be formulated to immediately address the determinants of MHL. This can be achieved by reviewing the existing MHL programs and campaigns to ensure that they are continuously being instituted at the general level and, more importantly, are targeted towards increasing the awareness of MHL among younger males, adolescents who indulge in substance abuse (i.e., smoking and alcohol consumption) as well as adolescents who are suffering from loneliness. Addressing the determinants of MHL may appear to some as a minor strategy in dealing with the overall burden of the mental health crisis. Nevertheless, it is a stepping stone in the direction to develop a generation of younger adolescents who are equipped with adequate MHL, which would subsequently enable them to maintain and sustain a healthy state of mental wellbeing.

## Figures and Tables

**Table 1:** Participant characteristics (*n* = 1400).

Characteristics		Frequency *n* (%)
Gender	Male	676 (48.3)
	Female	724 (51.7)
Age	13 years	687 (49.1)
	14 years	713 (50.9)
Ethnicity	Malay	928 (66.3)
	Non-Malay	472 (33.7)
Smoking status	Smoker	79 (5.6)
	Non-Smoker	1321 (94.4)
Consume alcohol	Yes	116 (8.3)
	No	1284 (91.7)
Been bullied	Yes	165 (11.8)
	No	1235 (88.2)
Felt lonely	Yes	566 (4.04)
	No	834 (59.6)
Parental marital status	Married and living together	1242 (88.7)
	Divorced/Separated	158 (11.3)
Parental income	Low income	970 (69.3)
	Moderate/high income	430 (30.7)
Intention to seek help	Yes	1139 (81.4)
	No	261 (18.6)
Correct recognition of depression	Yes	128 (9.1)
	No	1272 (90.9)
Adequate MHL	Adequate	122 (8.7)
	Inadequate	1278 (91.3)

**Table 2:** Factors associated with correct recognition of the mental health disorder (depression).

Variable	Univariate Logistic Regression		Multivariate Logistic Regression	
	Crude OR (95% CI)	*p*-Value	Adjusted OR (95%CI)	*p*-Value
Gender				
Male	1		1	
Female	1.57 (1.08, 2.28)	0.018 *	1.74 (1.17, 2.59)	0.006 **
Age				
13 years	1		1	
14 years	1.57 (1.08, 2.27)	0.018 *	1.54 (1.06, 2.24)	0.024 **
Ethnicity				
Malay	1		1	
Non Malay	0.72 (0.49, 1.05)	0.084 *	0.74 (0.49, 1.12)	0.160
Smoking status				
Smoker	1		1	
Non smoker	1.13 (1.03, 2.40)	0.044 *	1.61 (1.10, 3.57)	0.048 **
Consume alcohol				
Yes	1		1	
No	1.41 (1.78, 2.54)	0.026 *	1.31 (1.06, 2.53)	0.03 **
Been bullied				
Yes	1			
No	1.20 (0.66, 2.18)	0.549	-	-
Felt lonely				
Yes	1		1	
No	1.15 (1.06, 1.67)	0.040 *	1.22 (1.13, 1.79)	0.041 **
Parental marital status				
Married and living together	1		-	-
Divorce/Separated	0.88 (0.48, 1.60)	0.672		
Parental income				
Low	0.91 (0.61, 1.36)	0.642	-	-
Moderate/high	1			

Note. OR, Odds ratio; CI, Confidence interval; * Variables significant at 0.25 from the univariate analysis with no evidence of multicollinearity and interaction were entered into multivariate analysis. ** Significance set at *p*-value < 0.05 after multivariate analysis, 1 indicates a reference group. Hosmer-Lemeshow goodness-of-fit test chi-square = 9.656 (df = 7), *p* = 0.209. (Using enter method).

**Table 3:** Factors associated with intention to seek help.

Variable	Univariate Logistic Regression		Multivariate Logistic Regression	
	Crude OR (95% CI)	*p*-Value	Adjusted OR (95%CI)	*p*-Value
Gender				
Male	1		1	
Female	1.52 (1.16,1.99)	0.003 *	1.42 (1.06, 1.89)	0.018 **
Age				
13 years	1		1	
14 years	1.27 (1.02, 1.67)	0.006 *	1.28 (1.05, 1.69)	0.007 **
Ethnicity				
Malay	1		1	
Non Malay	0.83 (0.63, 1.10)	0.191 *	0.92 (0.67, 1.27)	0.627
Smoking status				
Smoker	1		1	
Non smoker	2.13 (1.30, 3.50)	0.003 *	1.68 (1.01,2.84)	0.045 **
Consume alcohol				
Yes	1		1	
No	1.93 (1.26, 2.95)	0.002 *	1.68 (1.03, 2.73)	0.037 **
Been bullied				
Yes	1		1	
No	1.36 (0.92, 2.01)	0.125 *	1.18 (0.79, 1.76)	0.425
Felt lonely				
Yes	1		1	
No	1.32 (1.01, 1.73)	0.043 *	1.32 (1.02, 1.75)	0.045 **
Parental marital status				
Married and living together	1		1	
Divorce/Separated	1.39 (0.94, 2.06)	0.103 *	1.36 (0.91, 2.03)	0.141
Parental income				
Low	0.97 (0.73, 1.31)	0.862	-	-
Moderate/high	1			

Note. OR, Odds ratio; CI, Confidence interval; * Variables significant at 0.25 from the univariate analysis with no evidence of multicollinearity and interaction were entered into multivariate analysis. ** Significance set at *p*-value < 0.05 after multivariate analysis, 1 indicates a reference group. Hosmer-Lemeshow goodness-of-fit test chi-square = 10.126 (df = 8), *p* = 0.256. (Using enter method).

**Table 4:** Factors associated with adequate MHL.

Variable	Univariate Logistic Regression		Multivariate Logistic Regression	
	Crude OR (95% CI)	*p*-Value	Adjusted OR (95%CI)	*p*-Value
Gender				
Male	1		1	
Female	1.49 (1.02, 2.18)	0.040 *	1.68 (1.12, 2.52)	0.012 **
Age				
13 years	1		1	
14 years	1.60 (1.09, 2.34)	0.015 *	1.56 (1.07, 2.30)	0.022 **
Ethnicity				
Malay	1		1	
Non Malay	0.71 (0.49, 1.04)	0.077 *	0.72 (0.47, 1.10)	0.126
Smoking status				
Smoker	1		1	
Non smoker	1.37 (1.17, 2.83)	0.017 *	1.99 (1.20, 4.26)	0.023 **
Consume alcohol				
Yes	1		1	
No	1.36 (1.14, 2.50)	0.032 *	1.23 (1.18, 2.41)	0.044 **
Been bullied				
Yes	1			
No	1.38 (0.728, 2.63)	0.323		
Felt lonely				
Yes	1		1	
No	1.19 (1.10, 1.73)	0.004 *	1.25 (1.06, 1.85)	0.027 **
Parental marital status				
Married and living together	1			
Divorce/Separated	0.76 (0.40, 1.45)	0.408		
Parental income				
Low	1.02 (0.69, 1.53)	0.914		
Moderate/high	1			

Note. OR, Odds ratio; CI, Confidence interval; * Variables significant at 0.25 from the univariate analysis with no evidence of multicollinearity and interaction were entered into multivariate analysis. ** Significance set at *p*-value < 0.05 after multivariate analysis, 1 indicates a reference group. Hosmer-Lemeshow goodness-of-fit test chi-square = 12.496 (df = 6), *p* = 0.052. (Using enter method).

**Figure A1:**
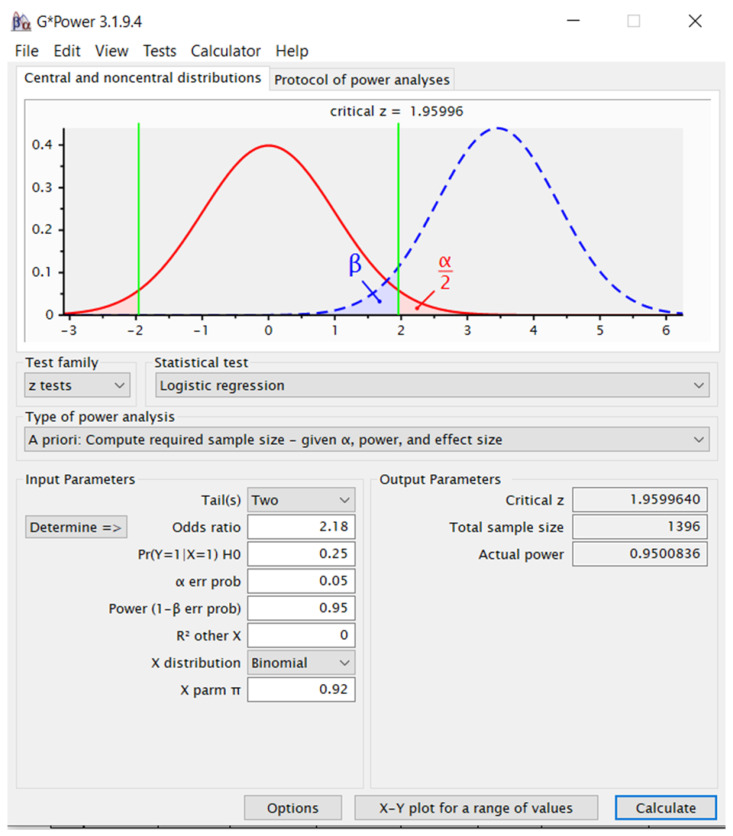
G* power sample size output.

## Data Availability

The data from this study are available in the supplementary file.

## Supplementary Materials

The following supporting information can be downloaded at: https://www.mdpi.com/article/10.3390/ijerph19063242/s1, Supplementary Materials S1: Data set.

## Appendix B. Test of Multicollinearity and Interaction

**Table A1:** Testing for multicollinearity among the independent variables significant at 0.25 in the univariate analysis examining the factors associated with correct recognition of depression/intention to seek help/adequate MHL.

	Gender	Age	Smoking	Alcohol	Felt Lonely
Gender		0.009	0.210	0.109	0.117
Age	0.009		0.048	0.011	0.033
Smoking	0.210	0.048		0.005	0.076
Alcohol	0.109	0.011	0.005		0.048
Felt lonely	0.117	0.033	0.076	0.048	

Note. Cramer’s V values of more than 0.3 indicate evidence of multicollinearity among variables.

**Table A2:** Testing for interaction among the independent variables significant at 0.25 in the univariate analysis examining the factors associated with correct recognition of depression/intention to seek help/adequate MHL.

**Variables in the Equation—Factors Associated with Correct Recognition of Depression**							
		**B**	**S.E.**	**Wald**	**df**	**Sig.**	**Exp(B)**
Step 1 ^a^	Age by Alcohol by Felt lonely by Gender by Smoking	16.030	6726.362	0.000	1	0.998 *	9156089.510
	Constant	2.291	0.093	609.990	1	0.000	9.883
^a^. Variable(s) entered on step 1: Age * Alcohol * Felt lonely * Gender * Smoking							
**Variables in the Equation—Factors Associated with Intention to Seek Help**							
		**B**	**S.E.**	**Wald**	**df**	**Sig.**	**Exp(B)**
Step 1 ^a^	Age by Alcohol by Felt lonely by Gender by Smoking	−0.480	0.246	3.797	1	0.051 *	0.619
	Constant	1.483	0.069	462.457	1	0.000	4.406
^a^. Variable(s) entered on step 1: Age * Alcohol * Felt lonely * Gender * Smoking							
**Variables in the Equation—Factors Associated with Adequate MHL**							
		**B**	**S.E.**	**Wald**	**df**	**Sig.**	**Exp(B)**
Step 1 ^a^	Age by Alcohol by Felt lonely by Gender by Smoking	15.978	6727.916	0.000	1	0.998 *	8689714.194
	Constant	2.344	0.095	611.362	1	0.000	10.418
^a^. Variable(s) entered on step 1: Age * Alcohol * Felt lonely * Gender * Smoking							

Note. * Non-significant value (*p* > 0.05) indicates no evidence of interaction.
